# Unforgettable in every way

**DOI:** 10.1002/jgf2.442

**Published:** 2021-05-04

**Authors:** Junki Mizumoto, Taro Shimizu

**Affiliations:** ^1^ Department of Medical Education Studies Graduate School of Medicine International Research Center for Medical Education The University of Tokyo Tokyo Japan; ^2^ Department of Diagnostic and Generalist Medicine Dokkyo Medical University Hospital Tochigi Japan

**Keywords:** internal medicine, polypharmacy

## 1 INTRODUCTION

An 86‐year‐old Japanese man presented with altered mental status (AMS) for several weeks and recent dyspnea and anorexia. He had been unwell for several weeks for an unknown reason, when 10 days before the presentation, he developed dyspnea, anorexia, and general malaise. In the late morning of the presentation day, when the patient saw his primary doctor on schedule, the doctor found him lethargic and referred him for further evaluation and treatment. The patient lived home with his wife. Per the patient's family and home‐visit care worker, the patient had a cognitive decline over the last several weeks, and he became to make frequent phone calls for help because he “does not know what to do.”

The patient is an older gentleman and therefore is at high vascular risk. Age‐related cellular immunodeficiency and malignancy risks also need to be mentioned. This patient's main problem is AMS that progressed in several weeks and dyspnea that started sometime later. AMS in this patient is primarily of content dysfunction rather than arousal dysfunction. Hence, a widespread cortical involvement should be considered.

Considering that dyspnea occurred in subacute AMS context involving diffuse cortical involvement, disorders with the respiratory control system stemming from central pathologies are concerned. I would list infectious or limbic encephalitis, Creutzfeldt‐Jakob disease (CJD), and hypothalamic‐pituitary‐adrenal (HPA) axis dysregulation, type A subacute aortic dissection affecting the anterior circulatory system with subsequent cardiac tamponade or aortic insufficiency, primary lung cancer with the anterior lobe metastasis, and ischemia on the anterior lobe of the brain. On the other hand, I also argue systemic pathologies affecting the cardiopulmonary system, the hematologic system, in search of the causes of dyspnea. These pathologies encompass metabolic disorders causing brain function and cardiac dysfunction (uremia, hypothyroidism, and syndrome of inappropriate antidiuresis (SIAD) based on intrathoracic pathology), toxic drug effect affecting both of central nervous and of cardiopulmonary vasculature, subacute infiltrative etiologies affecting multiple organs such as syphilis, HIV, lymphoma, or tuberculosis, indolent infective endocarditis (IE) with subsequent valvular destruction and systemic embolization, cardiac myxoma, and pulmonary tumor thromboembolic microangiopathy (PTTM). Meanwhile, since the patient is older, one may consider Hickam's dictum, an aphorism referring that a patient's clinical presentation is caused by more than one pathology: That is, AMS and dyspnea would be derived from different causes, respectively. Multiple medical conditions cause AMS with predominant content abnormality. Many of them are critical and require urgent diagnosis and intervention. Checking basic metabolic panel for abnormal blood glucose, uremia, and electrolyte abnormalities is the first test as it is quick and simple to pick up the critical and treatable condition. Other treatable causes of AMS include hypothyroidism, adrenal insufficiency, central causes such as encephalopathy, encephalitis, epilepsy, subdural hematomas, and depression, and insidious infectious diseases such as syphilis, tuberculosis, and HIV, and brain metastasis of the malignant neoplasm. All these conditions were already mentioned above.

The differential diagnoses of dyspnea alone in this patient include heart failure of any cause, pneumonia, pneumonitis, pulmonary embolism (arterial or venous), pneumothorax, pleural effusion of any cause, anemia of any cause, thoracic problems, neuromuscular diseases (paraneoplastic neuromuscular syndrome, phrenic nerve paralysis, and brainstem infarction), psychiatric conditions causing dyspnea (anxiety and panic disorders), or the compensation of metabolic acidosis.

The patient's past medical history included chronic obstructive pulmonary disease (COPD) with an episode of acute exacerbation 9 months before, pulmonary tuberculosis, chronic hepatitis C, hypertension, constipation, and insomnia. He had not had any history of pet or animal contact. He had not been exposed to any inhalant antigen as far as the family knew. He did not undertake home oxygen therapy because he did not show hypoxemia when taking a rest. He spent most of his day sitting on his chair or lying down on his bed and seldom went out or walked up and downstairs. His regular medication included tiotropium (2.5 µg/puff) 2 puff/d, slow‐release theophylline 400 mg/d, carbocisteine 1000 mg/d, verapamil 120 mg/d, magnesium oxide 660 mg/d, and zolpidem 10 mg/d. He seldom drank alcohol. He quitted smoking several days before.

The patient had COPD, usually because of smoking. Smoking is a significant risk factor for vascular diseases and malignant neoplasms, raising the possibility of these patients’ pathologies. It is interesting to note that he quitted smoking several days before. I would like to know what made the patient quit smoking. If he could not smoke because of his cognitive impairment or dyspnea, the patient's condition may indicate a severe condition. Malignancy‐related conditions and tuberculosis are of great concern. Paraneoplastic syndrome, lymphomas, lung or brain tumors, and PTTM should be ruled out. His tuberculosis history raises concerns about the reactivation of tuberculosis in the context of immunosuppression, causing dyspnea (pulmonary tuberculosis) and cognitive impairment (tuberculosis meningoencephalitis). In such an immobile patient with vascular risks, pulmonary embolism should be considered until proven otherwise. The patient also had a recent episode of acute COPD exacerbation. This medical history increased the possibility that the patient developed COPD exacerbation again. Triggers of COPD exacerbation include pneumonia and other infections, heart failure with or without acute coronary syndrome, pulmonary embolism, asthma, smoking, environmental factors, and drug nonadherence. However, there was no clinical information so far indicating preceding pneumonia, heart failure, pulmonary embolism, asthma, or substance inhalation before the development of dyspnea. Patients with COPD are vulnerable to hypercapnic narcosis resulting in a stupor, but it usually occurs in patients on oxygen therapy. Considering the patient's age and progressive cognitive decline, medication noncompliance may have been possible. If he recently forgot to inhale the powders or take medicines for COPD, he might develop dyspnea. If the patient failed to take verapamil for hypertension because of the cognitive decline, this might develop dyspnea from hypertensive heart failure. The patient may also be at risk of medication overuse because of cognitive decline. He was on theophylline, which is notorious for its potential toxicity. Theophylline poisoning in cognitively impaired older people is well known and common. It is also known that quitting smoking may elevate the theophylline concentration, sometimes leading to intoxication. The poisoning symptoms become severe and prolonged, especially with a single large dose or use of extended‐release agents. Adverse symptoms are diverse, affecting cardiac, pulmonary, metabolic, hematopoietic, and neuropsychiatric systems. Magnesium oxide may cause AMS due to hypermagnesemia, especially when a patient has chronic kidney disease. Zolpidem may also cause hypersomnolence. If the cognitive decline itself was due to medication poisoning, it is essential to check the prescription history. Besides, detailed history of the patient's current volume status is also necessary because medication intoxication is caused by elevated blood levels complicated by acute volume depletion, whatever the drug is.

The patient had chronic hepatitis C. If the patient's hepatitis developed into cirrhosis, hepatic encephalopathy with hepatopulmonary syndrome should be considered. The patient had constipation, one of the most common triggers of developing an elevation of ammonia. Cirrhosis is sometimes overlooked and underdiagnosed because it often lacks specific symptoms until uncompensated. Astute physicians may notice some characteristic physical signs, including palmar erythema, gynecomastia, spider angiomata, splenomegaly, nail and finger change (Muehrcke's nail, Terry's nail, clubbed finger, and hypertrophic osteoarthropathy), and testicular atrophy. His hypertension raises concerns about heart failure and aortic disorders (aortic dissection), which sometimes impair brain circulation, resulting in cognitive impairment in a subacute or chronic case. His constipation and insomnia might be from autonomic, metabolic, or endocrine dysregulation, including hypothyroidism, uremia, HPA axis dysfunction, or electrolyte disorders. The aforementioned other possibilities, including indolent IE, limbic encephalitis, CJD, presence of anemia, other intrathoracic pathologies, and neuropsychiatric disorders, should still be on the list.

On physical examination, the patient looked emaciated and lethargic with a Glasgow Coma Scale of 12 (E3, V4, M5). His body temperature was 37.0℃, blood pressure 128/70 mm Hg, pulse rate 88 bpm and regular, respiratory rate 28/min, and oxygen saturation 96% while breathing ambient air. There was no conjunctival pallor or icterus. Respiratory sounds were diffusely diminished, and fine crackles were heard on the upper chest bilaterally. No wheezes were heard. The intercostal space moved inwardly during inspiration. Heart sounds were normal without murmurs, rubs, and gallops. His abdomen was flat and soft, and there was no hepatosplenomegaly. His legs were not swollen without clubbing and cyanosis. The patient did not obey simple commands because of AMS. Pupillary light reflex was normal. Deep tendon reflexes were normal. Pathological reflexes, including Babinski's reflex, Hoffmann's sign, and Trömner's reflex, were not seen. There was no asterixis. Any apparent paralysis was not seen. There was no rigidity, coarse tremor, dystonia, or myoclonus.

On examination, the patient also had problems with arousal in addition to the problem of the contents. However, the differential listed so far does not change significantly at this moment. The patient's oxygen saturation seemed not too low despite his COPD. Tachypnea was the most prominent finding among his vital signs. The combination of seemingly normal oxygen saturation and tachypnea yields some clinical hypotheses. First, the patient may develop respiratory failure, and increasing respiratory rates may conceal hypoxemia due to pulmonary diseases (pneumonia and pneumonitis), pleural diseases (pleuritis, pneumothorax, and pleural effusion), or pulmonary vasculature disorders (pulmonary embolism and PTTM). Second, there may exist some extra‐pulmonary conditions causing tachypnea, including metabolic acidosis encompassing sepsis, uremia, drug intoxication, thoracic cage restriction due to pleural effusion, neuromuscular dysfunction, or pleurisy. Third, carbon monoxide intoxication may develop dyspnea with normal oxygen saturation.

The most notable finding on examination is lung sounds. Considering that the breath sounds were attenuated in almost all lung fields and crackles can be heard in both upper lung fields, there are several possibilities. First, it is reasonable to assume that most alveoli have been replaced by the liquid, except for a few alveoli in both upper lungs. The second likely interpretation is a situation in which both lung spaces are replaced by pleural fluid, and only the lungs of both upper lungs are functioning. Another possibility is that most of both lungs have emphysematous changes, and only both upper lungs are audible. Percussion is useful to differentiate whether the decreased breath sounds are due to liquid or air and should be performed after that. Chest retraction signs in this patient showed respiratory distress.

Heart sounds are normal, and therefore, it indicates that heart failure was less likely. There was no edema in the extremities, so elevated hydrostatic pressure (heart failure and volume overload) and findings of hypoproteinemia (cirrhosis, nephrosis, and cachexia) are not strongly suspected. Considering if massive pleural effusion exists in both lungs without anasarca, diseases limited to lung or pleura causing massive pleural effusion should be considered, such as tuberculosis, fungal infection, lymphoma, pleuritic malignancies, and chylothorax due to malignancy. If pleural effusion exists, thoracocentesis should be performed to examine the fluid. A computed tomography (CT) scan also clarifies the existence of mass lesions, pleural involvement, and other intrathoracic disorders, which are still not ruled out. Intravenous contrast CT will uncover vascular disorders, including pulmonary and aortic vessels, which are still concerning. Infective endocarditis should not be ruled out even without a heart murmur. Blood culture should be at least obtained. An echocardiogram is a good test of choice because this test elucidates valvular vegetation and regurgitation, pericardiac effusion, and sometimes dissecting flap in the aortic vessel.

Lacking in focal neurological findings lowers the likelihood of developing focal cerebral damages, including cerebral infarction and hemorrhage except for the frontal lobe involvement. Encephalitis and encephalopathy still should not be ruled out. Lumbar puncture should be performed if no other etiologies are explaining declined cognitive function. There seemed to be no evidence of anemia that would cause dyspnea. Since there was no jaundice appreciated, severe diseases of the hepatobiliary system were unlikely. Blood tests should rule out HPA axis dysfunction, hypothyroidism, and electrolyte dysfunctions. Adverse drug reactions are still concerning. Verapamil noncompliance is less likely because of his normal blood pressure.

Laboratory data (Table 1) revealed the white blood cell count of 9090/µL, hemoglobin level of 12.8 g/dL, platelet level of 31.8 × 10^4^/µL, serum creatinine level of 0.69 mg/dL, blood urea nitrogen level of 30.8 mg/dL, serum sodium level of 134 mEq/L, serum potassium level of 2.7 mEq/L, serum chloride level of 94 mEq/L, blood glucose level of 267 mg/dL, and serum D‐dimer level of 3.1 µg/mL (normal range, 0‐1.0 µg/mL). Aspartate aminotransferase level, alanine aminotransferase level, lactate dehydrogenase level, γ‐glutamyl transpeptidase level, creatinine kinase level, C‐reactive protein level, plasma ammonia level, and hemoglobin A1c level were within normal ranges. Arterial blood gas analysis data revealed a pH of 7.56, PaCO_2_ of 35.1 Torr, PaO_2_ of 110 Torr, and HCO_3_ of 31.5 mEq/L on ambient air. On urinalysis, neither pyuria nor bacteriuria was not seen. The electrocardiogram revealed a prolonged QT interval (QTc interval: 0.468 ms). Any arrhythmia was not seen. A chest CT scan showed emphysematous changes at the whole lung with reticular marking and bilateral slight pleural effusions, both of which had already been detected before. A head CT scan only showed mild atrophy of the frontal lobes. Transthoracic echocardiography revealed no abnormal findings.

**TABLE 1:** Laboratory data on admission.

		Normal range
WBC	9090/µL	3590‐9640
Hb	12.8 g/dL	13.2‐17.2
MCV	88.6	85.6‐102.5
PLT	31.8 × 10^4^/µL	14.8‐33.9 × 10^4^
AST	17 IU/L	8‐38
ALT	5 IU/L	4‐44
LDH	182 IU/L	106‐211
ɤ‐GTP	16 IU/L	14‐80
Cre	0.69 mg/dL	0.6‐1.1
BUN	30.8 mg/dL	7.0‐18.0
TP	7.1 g/dL	6.4‐8.2
Alb	3.0 g/dL	3.4‐5.0
CPK	79 IU/L	39‐308
Na	134 mEq/L	136‐145
K	2.7 mEq/L	3.5‐5.1
Cl	94 mEq/L	98‐107
Ca	8.9 mEq/L	8.5‐10.1
Glucose	267 mg/dL	74‐106
CRP	0.66 mg/dL	0‐0.30
Ammonia	52.0 µg/mL	30‐80
HbA1c	5.9%	4.5‐6.2
D‐dimer	3.1 µg/mL	0‐1.0
Arterial blood gas analysis data (breathing ambient air)		
pH	7.56	7.36‐7.44
PaCO_2_	35.1 Torr	36‐44
PaO_2_	110 Torr	80‐100
HCO_3_	31.5 mEq/L	22‐26
Base excess	8.9 mEq/L	−2.5‐2.5
Anion gap	11.2 mEq/L	10‐14
Urine analysis		
pH	6.0	
Protein	−	
Glucose	−	
Urobilinogen	±	
Occult blood	±	
Leukocyte	−	

Abbreviations: Alb, albumin; ALP, alkaline phosphatase; ALT, alanine aminotransferase; AST, aspartate aminotransferase; BUN, blood urea nitrogen; CPK, creatine phosphokinase; Cre, creatinine; CRP, C‐reactive protein; ɤ‐GTP, ɤ‐ glutamyl transpeptidase; Hb, hemoglobin; HbA1c, hemoglobin A1c; LDH, lactate dehydrogenase; MCV, mean corpuscular volume; PLT, platelet; T‐Bil, total bilirubin; TP, total protein; WBC, white blood cell.

Laboratory results showed low hemoglobin, normal kidney function, and normal liver enzyme, suggesting that the patient had no anemia, uremia, or hepatic encephalopathy. His blood glucose was high, but not high enough to explain the overall abnormality. The patient had no history of diabetes. If blood glucose rises despite the absence of diabetes, the common causes are acute physical stress and drug‐induced hyperglycemia with or without beta‐receptor stimulation. In the present case, it is not difficult to imagine that the patient is under acute stress, but as another possibility, theophylline's side effect should be investigated. Electrolytes were normal except for low potassium. Serum magnesium level was not obtained, it should have been obtained though. Hypokalemia causes intake loss, excessive loss from the gastrointestinal tract or urine, or intracellular shift. Checking urine potassium is the first step to determine the cause. Considering the patient's history, intake loss and elevated beta‐adrenergic activity with theophylline can be suspected. The high D‐dimer indicates intravascular thrombus formation, suggesting intravascular or systemic inflammation. His head CT scan was negative for hematoma or old stroke. There was an evidence of frontal lobe atrophy, which might have contributed to his cognitive decline. His chest CT revealed that the bilaterally diminished lung sounds were from emphysema, so the discussion of pleural effusion should be abandoned. At this point, there were no cytopenia, no abnormal lactate dehydrogenase level, little evidence of inflammation, and no explicit findings on imaging, making infiltrative diseases (lymphoma and tuberculosis) less likely. Other malignancies could not be ruled out completely, but there were no suggestive clinical findings.

His blood gas analysis unexpectedly showed metabolic alkalosis as the main component, probably from hypokalemia. There was concomitant respiratory alkalosis, which was probably due to tachypnea. The information I would need to know at this point includes history information suggesting psychiatric disorders, and history of medication compliance. I would suggest to add blood tests for thyroid function, cortisol, corticotropin, theophylline level, and blood culture. If these investigations do not reveal the cause, lumbar puncture and contrast‐enhanced magnetic resonance imaging were performed for detecting intracranial lesions that cannot be detected by noncontrast head CT. Besides, a whole‐body contrast‐enhanced CT with or without 18F‐fluorodeoxyglucose positron emission tomography for neoplastic lesions or intravascular lesions may be considered.

Based on his symptoms, data, and a history of taking theophylline, he was tentatively diagnosed with theophylline intoxication. Two sets of blood cultures were obtained to rule out bacteremia. The patient did not develop seizures or significant electrocardiography changes, and administration of activated charcoal or hemodialysis was not performed. Fluid repletion with potassium was initiated, and theophylline intake was discontinued. The patient was admitted for further evaluation and treatment. Additional diagnostic tests were planned to perform if the patient's AMS did not improve even on the next day.

The patient's constellation of symptoms including general malaise, loss of appetite, cognitive decline, tachypnea with acute respiratory alkalosis, hypokalemia, and hyperglycemia was attributed to theophylline intoxication, possibly triggered by recent quitting of smoking. Declining cognitive impairment might also have accelerated to worsen the medication misusage, resulting in the poisoning. For the management of theophylline intoxication, the strategy of lowering the circulating concentration of theophylline includes gastrointestinal decontamination (an administration of activated charcoal 1 g/kg by mouth or nasogastric tube) and hemodialysis. Most intoxicated patients are, however, successfully managed with supportive care. The patient should be kept in close monitoring for at least 2 days because the half‐time of slow‐release theophylline is about 15 hours in healthy young adults.

On the second day in the hospital, serum theophylline level showed 44.2 µg/mL (reference range, 5.0‐15.0 µg/mL). This level was high enough to develop various symptoms due to theophylline toxicity. Bacterial culture turned out to be negative. On the third day, the patient's mental status and respiratory rates turned utterly normal, and he did not complain of dyspnea or general malaise. His cognitive decline was also improved. An electrocardiogram revealed a normal QT interval. The patient was undertaken in‐hospital rehabilitation. On day 29, the patient was discharged to the nursing home, where nursing staff helped him inhale a long‐acting beta‐agonist plus long‐acting muscarinic antagonist.

The chronology of this case is as follows. Several weeks before, chronic mild intoxication caused cognitive decline and feeling unwell. Ten days before, dyspnea and anorexia were developed and the patient quitted smoking, which unfortunately increased theophylline concentration to over 40 µg/mL. In the subsequent few days, acute exacerbation on chronic intoxication occurred and AMS was developed. This time course is also supported by the findings of hypokalemia, hyperglycemia, and respiratory alkalosis, which were more typical in acute theophylline poisoning rather than chronic.

## 2 DISCUSSION

This case illustrates an older patient with AMS, tachypnea without hypoxemia, hypokalemia, and hyperglycemia. These findings turned out to be a manifestation of theophylline intoxication. The patient's cognitive decline was improved after stopping theophylline intake. In most of the healthcare facilities, it takes several times to turn out serum theophylline concentration. Suspecting theophylline intoxication based on history taking and nonspecific systemic abnormalities is therefore important.

Dyspnea is “the result of a complex interaction of physiological, psychosocial, social, and environmental factors.”^1^ In this case, dyspnea was probably caused by multiple factors, including tachypnea due to theophylline intoxication, AMS, and other psychological disturbance due to intoxication, baseline COPD condition, and growing anxiety of the patient and his wife.

Theophylline was formerly used for asthma and COPD because it antagonizes adenosine receptors and functions as a bronchodilator. Its various side effects lie in the wide distribution of adenosine receptors throughout the body.^2^ Besides, theophylline also activates catecholamine release indirectly.^2^ Because of its side effects and a broad acceptance of inhaled beta‐agonist and muscarinic antagonist, theophylline now plays a minimal role in treating asthma^3^ or COPD.^4^ However, theophylline is still widely used to treat asthma and COPD, especially in developing countries.^5^ Japan is another country in which theophylline is widely prescribed. One study revealed that about 15% of patients with asthma were prescribed theophylline.^6^ The therapeutic concentration of theophylline for asthma or COPD ranges from 10 to 20 µg/mL. Unfortunately, theophylline has a very narrow therapeutic ratio, and poisoning symptoms or signs may develop at the serum concentration of 20 µg/mL. It is typically said that serum concentrations of 80‐100 µg/mL in acute overdoses and 40‐60 µg/mL in chronic overdoses are associated with life‐threatening toxicity.^7^ Theophylline concentration may fluctuate by various factors, one of which is smoking. Theophylline is a hepatic cytochrome P‐450 (CYP) isoenzyme 1A2 substrate. Tobacco smoke contains polycyclic aromatic hydrocarbons, potent inducers of CYP 1A2. Quitting smoking rapidly decreases CYP1A2 and increases theophylline concentration,^8^ as in this case. Physicians should know theophylline intoxication presentation and treatment, even if ones do not prescribe this drug regularly. Theophylline is a drug that is “unforgettable in every way,” like a jazz masterpiece.

Adverse drug reaction is common in older patients, and physicians should always keep iatrogenic conditions in mind when seeing older patients. Using STOPP (Screening Tool of Older Persons’ Prescriptions) and START (Screening Tool to Alert to Right Treatment) criteria is one of the various strategies for preventing adverse drug reactions, and these criteria may be useful in improving clinical outcomes in multimorbid older people.^9^ STOPP/START criteria say that theophylline as monotherapy for COPD should not be prescribed.^10^ If the patient had received a medication review, this adverse drug reaction might have been prevented.

In summary, this case conveys three important messages. First, physicians should always suspect adverse drug reactions when seeing older patients taking multiple medications, taking any potentially inappropriate medication, or manifesting multiple‐organ dysfunction. Physicians should be aware that two “reviews” are important to correct diagnoses, especially seeing older patients: review of systems and review of prescription. Second, as theophylline intoxication syndrome manifests diverse symptoms, patients taking theophylline with any complaints should be at least once suspected of developing the intoxication. In particular, tachypnea without hypoxemia and slight AMS may be easily overlooked unless physicians do not keep theophylline toxidrome in mind. Detailed history information is also important because smoking cessation may lead to theophylline poisoning. Third, theophylline should not be used in frail older patients. Theophylline is now not strongly recommended for patients with asthma or COPD. Physicians who prescribe theophylline for some inevitable reasons should monitor the concentration regularly and pay close attention to drug interaction including tobacco.

## CONFLICT OF INTEREST

The authors have stated explicitly that there are no conflicts of interest in connection with this article.
